# Combining degree centrality and betweenness centrality of molecular networks can effectively pinpoint individuals at high risk of HIV transmission within the network

**DOI:** 10.3389/fcimb.2025.1695049

**Published:** 2026-01-19

**Authors:** Wen Gan, Bin Zhao, Wei Song, Mingming Kang, Xue Dong, Xin Li, Lu Wang, Jianmin Liu, Haibo Ding, Zhenxing Chu, Lin Wang, Wen Tian, Hong Shang, Xiaoxu Han

**Affiliations:** 1 State Key Laboratory for Diagnosis and Treatment of Infectious Diseases, National Clinical Research Center for Laboratory Medicine, The First Hospital of China Medical University, China Medical University, Shenyang, China; 2 NHC Key Laboratory of AIDS Prevention and Treatment, National Clinical Research Center for Laboratory Medicine, The First Hospital of China Medical University, China Medical University, Shenyang, China; 3 Key Laboratory of AIDS Immunology, Chinese Academy of Medical Sciences, Shenyang, Liaoning, China; 4 Department of Food Safety and Nutrition, Shenyang Center for Health Service and Administrative Law Enforcement (Shenyang Center for Disease Control and Prevention), Shenyang, China

**Keywords:** betweenness centrality, degree centrality, HIV, men who have sex with men (MSM), molecular network

## Abstract

**Introduction:**

HIV molecular network technology can identify HIV transmission hotspots and individuals at risk of HIV transmission, facilitating precise and targeted interventions. This study explored the molecular network parameters, namely degree centrality (DC) and betweenness centrality (BC), to effectively pinpoint individuals at high risk of HIV transmission within the network.

**Methods:**

A previous whole-population sampling cohort comprising all newly diagnosed people living with HIV (PLWH) in Shenyang, from 2016 to 2019, was analyzed. Molecular networks based pol gene were constructed, the DC and BC of each node were calculated, and six groups of nodes were identified based on DC, BC, and DC+BC: high DC group, low DC group, high BC group, low BC group, high DC+BC group, and non-high DC+BC group. The average risk of HIV transmission in each group was calculated by dividing the total probability of recent HIV infection (identified by HIV-1 LAg-Avidity EIA) by the number of cases in each group. A multivariate logistic regression analysis was conducted to identify the characteristics of the high-risk group.

**Results:**

Of the 2882 PLWH, 1162 were included in the molecular network. The mean DC and the mean BC of all nodes were 2.6 (range: 1-29) and 0.09 (range: 0-1), respectively. The top three groups with the highest average risk of HIV transmission were the high DC+BC group at 0.62, followed by the high BC group at 0.56, and the high DC group at 0.53. The characteristics of the high DC+BC group were low education levels, Housekeeping, housework, and unemployment, and high baseline viral load (≥10^5^copies/mL) (P<0.05).

**Discussion:**

The combined utilization of DC and BC can effectively identify individuals at high risk of HIV transmission, enabling precisely targeted interventions using molecular network technology.

## Introduction

The Joint United Nations Programme on HIV/AIDS has set the ambitious goal of ending the HIV epidemic by 2030 and emphasizing the “three 95%” strategy (UNAIDS, 2015). As of 2021, 85% of people living with HIV (PLWH) worldwide were aware of their HIV status. Among those aware, 88% had access to antiretroviral therapy (ART), and among those on ART, 92% had achieved viral suppression (UNAIDS, 2025). Recent studies conducted in Europe and China have revealed that undiagnosed PLWH were the primary drivers of sustained HIV transmission (Ratmann et al., 2016; Zhao et al., 2022). Therefore, a key aspect of achieving the “three 95%” goal and putting an end to the global HIV epidemic is to increase the diagnosis rate of undiagnosed PLWH and provide early access to ART.

The emerging HIV molecular network technology based on the HIV pol sequence can reconstruct the HIV transmission history and reveal the transmission links among HIV-infected individuals (Smith et al., 2009; Oster et al., 2015; Hassan et al., 2017). This technology holds significant importance in identifying HIV transmission hotspots and individuals at risk of HIV transmission, allowing targeted interventions to focus on high-risk populations and undiagnosed PLWH (Little et al., 2014). Degree centrality (DC, number of links) is commonly used as a parameter, and high DC indicates influence within the network (Mazrouee et al., 2022). Most studies suggested that nodes in the network with more links had a higher risk of transmitting HIV, and targeting these nodes for intervention can be more effective (Oster et al., 2015; Wang et al., 2015; Morgan et al., 2017). In many studies, nodes with a DC >3 or 4 were defined as individuals at high risk of HIV transmission (NATIONAL CENTER FOR AIDS/STD CONTROL AND PREVENTION, 2019; Zhang et al., 2020; Jiang et al., 2022; Zhao et al., 2022). However, DC may not truly reflect the time-varying transmission risk of individuals within the network, because the DC of individuals within the network only increases with the increase of individuals entering the network, and does not decrease over time.

Betweenness centrality (BC) is another important parameter in social network analysis (SNA), which measures the ability of a node in the network to act as a “medium” or a “bridge”. BC quantifies the number of shortest paths between pairs of nodes that pass through the node being examined. Nodes with the shortest paths passing through them have higher BC values. BC relies more on the individual’s position in the network, rather than simply connecting to evaluate their risk in the network. BC has been utilized to target the bridge population that plays a significant role in HIV transmission across different social circles. It has also been used to assess the importance of non-disclosed men who have sex with men (MSM) in molecular networks (Tieu et al., 2015). Hence, relying solely on DC as a measure of HIV transmission risk may not provide a comprehensive assessment, potentially overlooking the significance of the “medium” or “bridge” individuals within the network. However, no studies have been conducted to establish a correlation between individuals with higher BC in the network and a higher risk of HIV transmission.

Shenyang is the capital of Liaoning Province and the center of Northeast China, with a permanent population of 9.204 million as of 2023. In 2019, the number of PLWH in Shenyang had reached 7,000 (Zhao et al., 2021b), of which MSM accounted for more than 80% of PLWH (Zhao et al., 2021a). In this study, a whole population-based molecular network was established in Shenyang from 2016 to 2019, aiming to make full use of existing network parameters to effectively identify individuals at high risk of HIV transmission within the molecular network. The study’s findings provided a more comprehensive analytical strategy to guide effective and precise targeted interventions using molecular network technology.

## Materials and methods

### Study population

A comprehensive retrospective cohort study was conducted in Shenyang, China, from January 1, 2016, to December 31, 2019 (Zhao et al., 2021a, 2021). Baseline demographic information, including age at the time of diagnosis, gender, ethnicity, marital status, education and occupation, infection route, and diagnosis date, was collected. Clinical data such as baseline virus load (VL), baseline CD4+ T cell count, and the results of recent HIV infection (RHI, identified by HIV-1 LAg-Avidity EIA) were also obtained. Additionally, the HIV pol sequences (HXB2: 2268-3302) and the data of HIV drug-resistant mutations were also collected and have been publicly available (Zhao et al., 2021b). The study was approved by the Institutional Review Board of China Medical University.

### Inferring molecular network

Briefly, all paired genetic distances (GD) between sequences were calculated using the Tamura-Nei 93 model. The molecular networks for three main prevalent subtypes (CRF01_AE, CRF07_BC, and B) were built based on the optimal genetic distance (GD) threshold by using the HIV-TRACE (Kosakovsky Pond et al., 2018). GD threshold sensitivity analysis has been conducted to ensure that the molecular network constructed using the obtained optimal GD threshold can generate the most molecular transmission clusters (Zhao et al., 2021a, 2021). A cluster was defined as a connection component in the network consisting of at least two nodes. The molecular network visualization was generated using Cytoscape v3.8.2 (Shannon et al., 2003).

### Calculation of DC and BC and grouping

The DC and BC of all nodes in the network were calculated. The DC of each node was determined by counting the number of links it had. BC was calculated using the formula of $\text{BC}\left(i\right)=\sum_{j\neq k\neq i}^{N}\frac{g_{jk}\left(i\right)}{g_{jk}}/\frac{\left(N-1\right)\left(N-2\right)}{2}$. Here, $g_{jk}$ represents the number of all shortest paths from node j to node k, and $g_{jk}\left(i\right)$ represents the number of paths through node i in these paths. N represents the total number of nodes in the network. The BC value ranges from 0 to 1, with a higher value indicating that the node has a greater capacity to act as a bridge within the network.

Six groups were established based on DC, BC, and the combination of DC+BC. 1. high DC group: individuals with a DC value greater than the mean; 2. low DC group: individuals with DC value less than or equal to the mean; 3. high BC group: individuals with BC value greater than the mean; 4. low BC group: individuals with BC value less than or equal to the mean; 5. high DC+BC group: individuals with both high DC and high BC; 6. non-high DC+BC group: individuals who do not have both high DC and high BC simultaneously.

### Risk assessment for HIV transmission

Usually, the links within a molecular network have no inherent directionality, as it is often impossible to confirm the exact timing of HIV infection for individuals in the network. However, in this study, we collected the diagnosis time of newly diagnosed PLWH as well as RHI results. This information allowed us to infer the HIV transmission direction among the interconnected individuals in the network. By quantifying the RHI that may be caused by individuals in different groups, we were able to estimate the overall risk of HIV transmission posed by each group within the network. The risk of HIV transmission for each group was measured by examining the number of RHI within the network caused by each group. The average risk of HIV transmission between different groups was calculated and compared based on their contribution to RHI.

**Figure 1:**
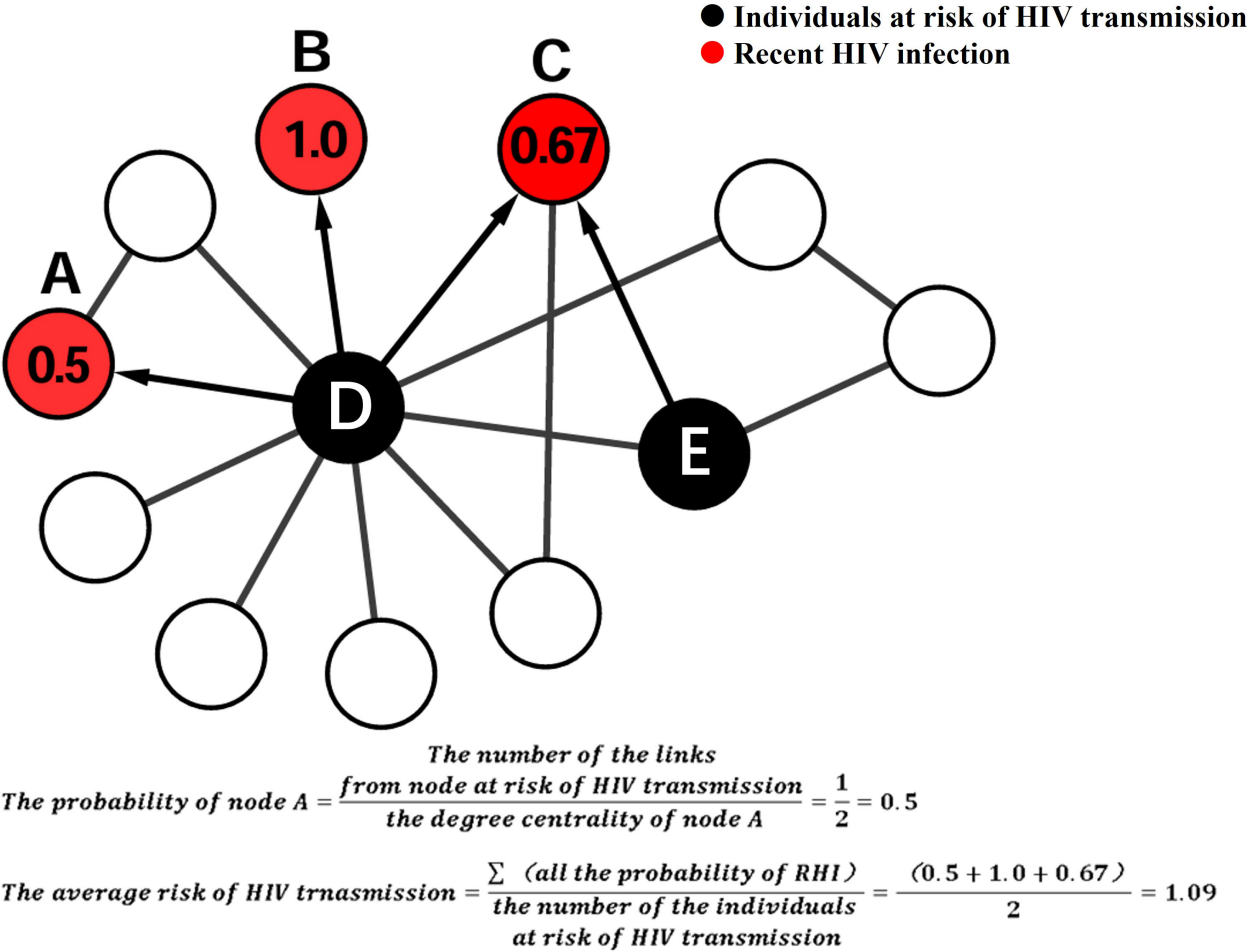
Schematic illustrating the calculation of average HIV transmission risk within a molecular cluster.

In this study, RHI within the network were considered as index cases, while individuals linked to index cases were considered as potential sources of infection. The direction of HIV transmission between potential sources of infection and index cases was determined based on HIV infection status (RHI and CHI) and the timing of HIV diagnosis. For example, The HIV infections contributing to the RHI in 2018 can only come from CHI who were infected with HIV before 2018 (inclusive), or RHI who were diagnosed with HIV earlier than him in 2018 (Zhao et al., 2022) If an RHI in the network has N links, with k of those links identified from individuals at high risk of HIV transmission, the probability of RHI becoming infected through these individuals at high risk of HIV transmission was calculated as k/N. In **Figure 1**, nodes A, B, and C were RHI, and their DC of them were 2, 1, and 3, respectively. The probability of nodes A, B, and C being infected through these individuals at risk of HIV transmission (nodes D and E) was 0.5(1/2), 1.0(1/1), and 0.67(2/3), respectively. The average risk of HIV transmission was calculated by dividing the total probability of RHI by the number of individuals at risk of HIV transmission. The average risk of HIV transmission of nodes D and E was 1.09([0.5 + 1.0 + 0.67]/2).

### Statistical analyses

Multivariate logistic regression analysis was conducted to determine the characteristics of MSM, with odds ratio (OR), adjusted odds ratio (AOR), and 95% confidence intervals (CI). In the univariate analysis model, independent variables with a p-value<0.1 were included in the multivariable model. P-value<0.05 was considered statistically significant. The statistical analysis was performed using SPSS version 25.0.

## Results

### Study population

A total of 2882 newly diagnosed HIV-infected individuals with available pol sequences were obtained in Shenyang from 2016 to 2019. Among the participants, 93.2% (2686/2882) were male, with a median age of 32 years (interquartile range: 26–45 years, range: 1–89 years). The majority, 82.1% (2,367/2,882), identified as MSM. Furthermore, 70.0% (2019/2882) had a senior high school education or above, and 75.0% (2162/2882) were housekeeping, housework and unemployment. At the time of HIV diagnosis, 33.1% (953/2882) were identified as RHI. The mean baseline CD4+T cell count was 305 ± 209 cells/μl (n=1986) and the mean baseline VL was 4.7 ± 0.7 log10 copies/ml (n=1577). (**Table 1**). CRF01_AE (70.0%, 2019/2882), CRF07_BC (18.3%, 526/2882), and subtype B (4.6%, 132/2882) were the main epidemic strains in Shenyang. 9.7%(279/2882) had drug-resistant mutations.

**Table 1:** Characteristics of the individuals in this study.

Characteristics	Total(N)	%
Total	2882	100
Gender		
Female	196	6.8
Male	2686	93.2
Age		
≤25	653	22.7
26-45	1531	53.1
≥46	698	24.2
Ethnicity		
Han	2482	86.1
Others	400	13.9
Marital status		
Married	548	19.0
Unmarried	1818	62.7
Divorced	519	18.0
Not available	7	0.3
Education		
Senior high school and above	2019	70.1
Junior high school and below	853	29.6
Not available	10	0.3
Occupation		
Housekeeping, housework and unemployment	1910	66.3
Employed and retirees	774	26.8
Others/Not available	198	6.9
Infection Route		
Men who have sex with men	2367	82.1
Heterosexual transmission	434	15.1
Injection drug users	44	1.5
Other/Not available	37	1.3
Number of sexual partners		
<10	1608	55.8
≥10	188	6.5
Not available	1086	37.7
Subtype		
CRF01_AE	2019	70.0
CRF07_BC	526	18.3
B	132	4.6
Other	205	7.1
Drug-resistant mutations		
No	2603	90.3
Yes	279	9.7
Infection status		
Recent HIV infection	953	33.1
Chronic HIV infection	1868	64.8
Not available	61	2.1
CD4+T cells (cells/µL)	305 ± 209(n=1986) 4.7 ± 0.7(n=1577)	
Viral Load ((log10 copies/mL)		

### Molecular network analyses and determination of cutoff values

**Figure 2:**
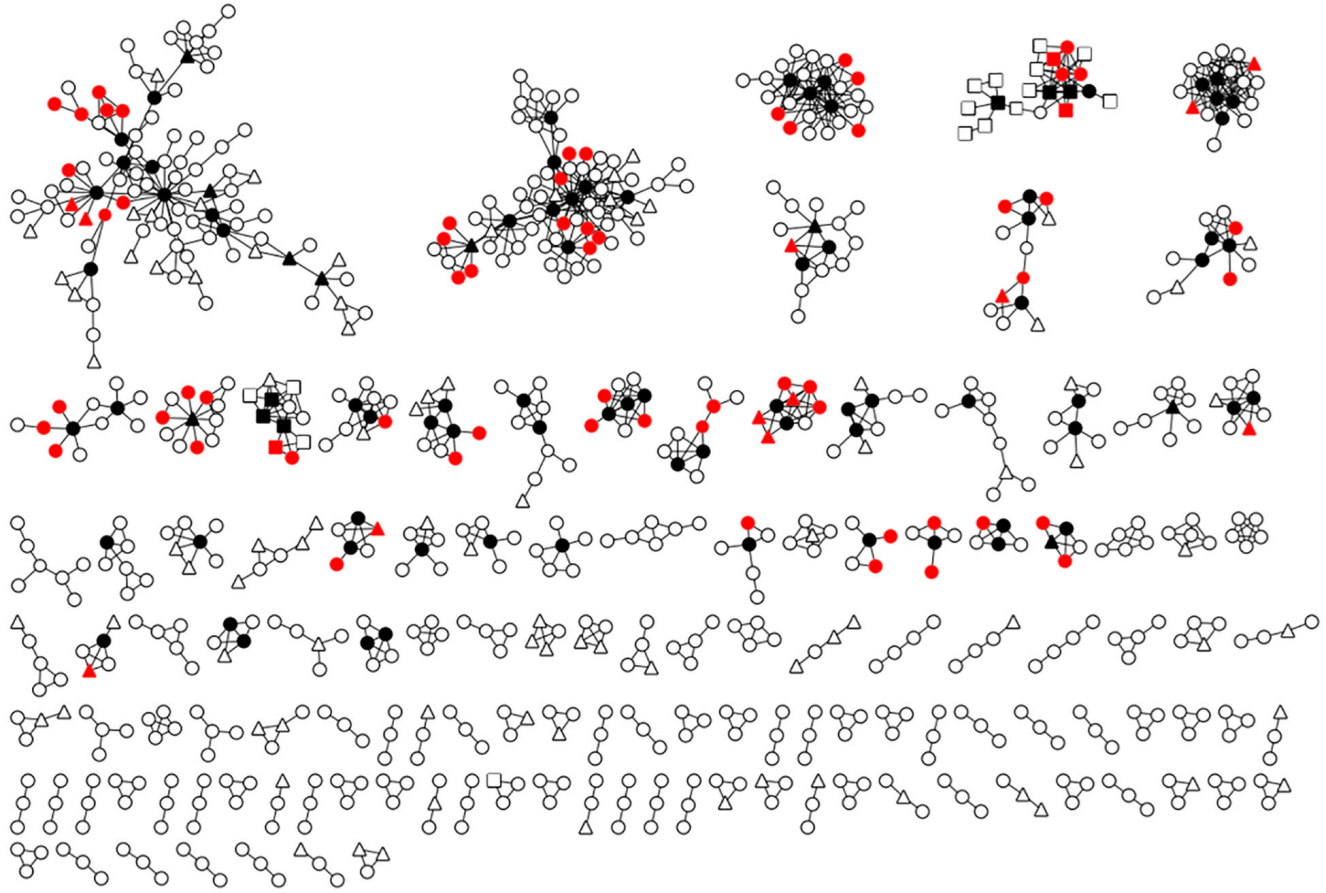
The molecular transmission network and average HIV transmission risk associated with the high DC+BC group. Black nodes denoted the individuals within the high DC+BC group. Red nodes denoted recent HIV infection caused by the high DC+BC group. Circular nodes represented men who have sex with men, triangular nodes represented the Heterosexual transmission and square nodes represented the injection drug user.

The network consisted of 305 clusters and 1162 nodes, including 232 clusters for CRF01_AE (size: 2-99, 857 nodes), 55 clusters for CRF07_BC (size: 2-72, 252 nodes), and 18 clusters for subtype B (size: 2-8, 49 nodes) (**Figure 2**). The mean DC of all nodes in the network was 2.6 (range: 1-29), and the mean BC of all nodes was 0.09 (range: 0-1). The distribution of DC and BC of all nodes within the network was skewed. 50.8% of nodes (590/1162) had a DC of 1, and 74.7% of nodes (868/1162) had a BC of 0. There was a weak linear correlation between DC and BC (r=0.25, P<0.05).

**Figure 3:**
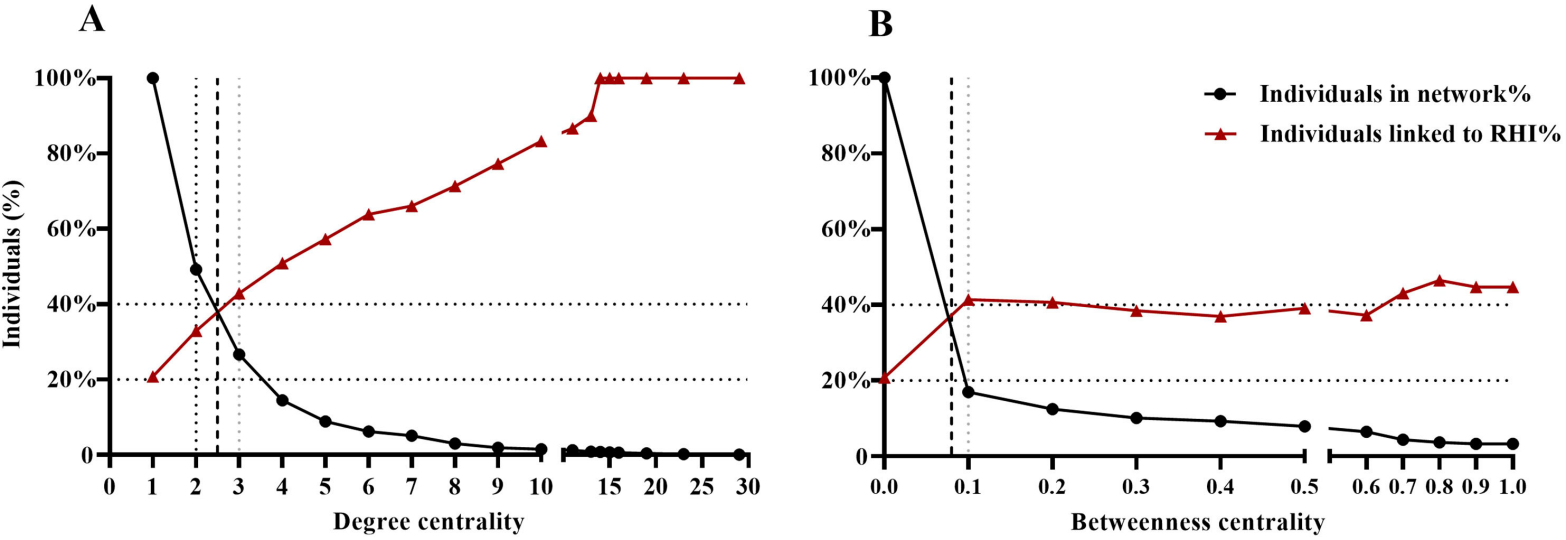
Sensitivity analysis for determining cutoff values of degree centrality **(A)** and betweenness centrality **(B)** to identify high-risk individuals.

Subsequently, we explored the cutoff values of DC and BC to identify high-risk individuals. As DC increased from 1 to 29, the proportion of individuals with high DC in the network gradually decreased (100.0% to 0.1%). In contrast, the proportion of the high-risk individuals capable of causing RHI gradually increased (from 20.8% to 100.0%), indicating that individuals with higher DC could have a higher risk of transmission in the network. The two curves intersected at a DC value of 2.5 (**Figure 3A**). Similarly, in the BC cutoff analysis, the intersection occurred at a BC value of 0.08 (**Figure 3B**). Given that the mean values of DC (2.6) and BC (0.09) were very close to these intersection points and were more readily applicable, we adopted them as the cutoff thresholds for classifying high-risk individuals. Based on these cutoffs, six distinct groups were defined: high DC (n=169), low DC (n=993), high BC (n=201), low BC (n=961), high DC+BC (n=92), and non-high DC+BC (n=1070).

In this study, we further assessed the performance of DC and BC in identifying high-risk individuals as the molecular network evolved annually. Our analysis revealed that although the mean values of DC and BC remained relatively stable over time, the proportion of high-risk individuals associated with RHI increased significantly (DC: P<0.001, BC: P = 0.004; **Supplementary Figures 1A, B**).

### Analysis of HIV transmission risk

**Figure 4:**
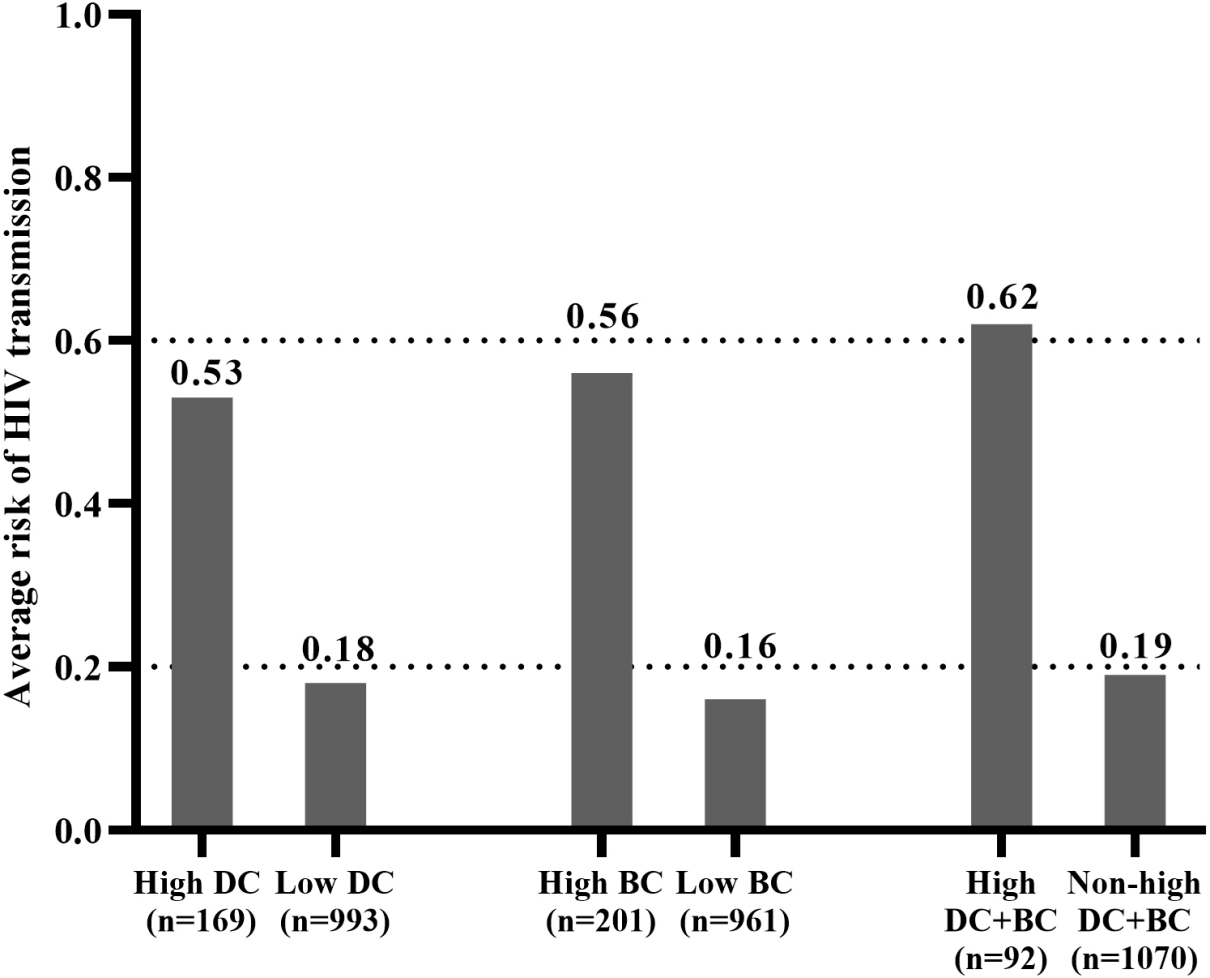
Estimated average risk of HIV transmission in the six groups.

The total probability of RHI was first calculated for six groups, revealing that the high DC group contributed 89.86 RHI, the low DC group contributed 176.34 RHI, the high BC group contributed 113.22 RHI, the low BC group contributed 152.98 RHI, and High DC+BC group contributed 57.4 RHI, and the non-high DC+BC group contributed 208.8 RHI. Subsequently, the average risk of HIV transmission was calculated, with the highest risk observed in High DC+BC group (0.62) (**Figure 4**), followed by high BC group (0.56), high DC group (0.53), non-high DC+BC group (0.19), low DC group(0.18), and low BC group (0.16) (**Figure 4**). These findings suggested that interventions targeting the high DC+BC group may be the most effective in reducing HIV transmission.

### Characteristics of MSM at high risk of HIV transmission

To minimize the introduction of additional confounding factors (other HIV infection routes), our analysis focused solely on MSM, as they accounted for more than 80% of PLWH in Shenyang (82.1% within this cohort). We analyzed the characteristics of the top three groups of MSM with the highest intervention efficiency, namely the high DC group, the high BC group, and the high DC+BC group. In comparison to other MSM (MSM outside the network and MSM without high risk of HIV transmission within the network), the characteristic of MSM in the high DC group was having a junior high school education or below (AOR = 1.882, P = 0.001), CRF07_BC subtype infection (AOR = 2.079, P<0.001), and carrying drug-resistant mutations (AOR = 1.991, P = 0.004). For MSM in the high BC group, the characteristics were Housekeeping, housework, and unemployment (AOR = 1.749, P = 0.010), and having a high baseline VL (≥10^5^copies/mL AOR = 2.602, P<0.001). The characteristics of MSM in the high DC+BC group included having a junior high school education or below (AOR = 1.733, P = 0.023), Housekeeping, housework, and unemployment (AOR = 1.875, P = 0.049), and having a high baseline VL (AOR = 2.994, P = 0.002) (**Table 2**).

**Table 2:** The characteristics of MSM in high DC group, high BC group and high DC+BC group.

Risk factors	Total (N, %)	MSM^a^ with high DC^b^ (N, %)	Multivariate analyses				MSM with high BC^e^ (N, %)	Multivariate analyses				MSM with high DC+BC (N, %)	Multivariate analyses			
			AOR^c^	95% CI^d^	P-value	H-L test^f^		AOR	95% CI	P-value	H-L test		AOR	95% CI	P-value	H-L test
**Total**	2367(100.0)	135(5.7)				**P=0.299**	168(7.1)				**P=0.971**	76(3.2)				**P=0.827**
**Education**																
Senior high school and above	1765(74.6)	48(2.7)		**Ref**								45(2.6)		**Ref**		
Junior high school and below	598(25.3)	86(14.4)	1.882	1.296-2.732	**0.001**							31(5.2)	1.733	1.077-2.788	**0.023**	
Not available	4(0.2)	1(0.25)										0(0.0)				
**Occupation**																
Housekeeping, housework and unemployment	1578(66.6)						122(7.7)	1.749	1.141-2.681	**0.010**		58(3.7)	1.875	1.002-3.507	**0.049**	
Employed and retirees	624(24.5)						31(5.0)		**Ref**			15(2.4)		**Ref**		
Other/Not available	165(6.4)						15(9.1)					3(1.8)				
**Viral Load (copies/mL)**																
<10**^5^**	921(38.9)						36(3.9)		**Ref**			14(1.5)		**Ref**		
≥10**^5^**	420(17.7)						41(9.8)	2.602	1.588-4.263	**<0.001**		20(4.8)	2.994	1.481-6.053	**0.002**	
Not available	1026(43.3)						91(8.7)					42(4.1)				
**Subtype**																
CRF01_AE	1655(70.0)	87(5.3)		**Ref**												
CRF07_BC	458(19.3)	47(10.3)	2.079	1.438-3.059	**<0.001**											
B	102(4.3)	1(1.0)	6.110	0.840-44.461	0.074											
Other	152(6.4)	–														
**Drug-resistant mutations**																
No	2130(90.0)	110(5.2)		**Ref**												
Yes	237(10.0)	25(10.5)	1.991	1.250-3.169	**0.004**											

a men who have sex with men

b degree centrality

c Adjusted Odds Ratio

d Confidence Interval

e betweenness centrality

f Hosmer-Lemeshow. The values with statistical significance (p < 0.05) were presented in bold.

## Discussion

In this study, we constructed a city-level HIV molecular transmission network and explored the effectiveness of molecular network parameters in identifying individuals at high risk of HIV transmission. Our findings revealed that the combined utilization of DC and BC can effectively identify individuals at high risk of HIV transmission while providing a more comprehensive understanding of their characteristics.

The primary objective of this study was to explore the effectiveness of molecular network indicators (DC and BC) in identifying individuals at high risk of HIV transmission. A previous study predicted the risk of HIV transmission among the individuals within the network by analyzing the associations of baseline high-risk behaviors, such as the number of unique sexual partners and insertive unprotected anal intercourse (Little et al., 2014). In another study, the transmission rate (TR) of molecular clusters was calculated using Bayesian molecular clock phylogenetic inference to estimate the HIV transmission efficiency within large molecular clusters, revealing a median TR of 52.4 per 100 person-years for eight large clusters (Zhao et al., 2021a). The above-mentioned methods indirectly assessed the risk of HIV transmission for individuals or molecular clusters within a network. In contrast, our study directly evaluates the HIV transmission risk by quantifying the contribution of individuals in a network to HIV infection. At the same time, given limited resources, interventions become more cost-effective when a larger number of HIV infections can be prevented by targeting a smaller number of key individuals. Our study revealed that individuals in the high DC+BC group exhibited the highest average risk of HIV transmission among six groups, suggesting that targeting interventions towards individuals with high DC and BC may be the most effective approach.

Our results also emphasized the potential benefits of the combined utilization of DC and BC to achieve a more comprehensive understanding of the characteristics associated with individuals at risk of HIV transmission. Our findings revealed that among MSM in the high DC group, a notable characteristic was having a junior high school education or below. This finding aligns with a recently published systematic review that reported a higher HIV prevalence among the illiterate population (16.8%) compared to those with an education in China (Dong et al., 2019). Additionally, another recent research highlighted that male sex workers (MSWs) in China tend to have lower levels of education (Yu et al., 2022). On the other hand, our study found that unemployment emerged as a risk factor for MSM with high BC. This finding was consistent with the fact that MSWs often faced unemployment and engaged in sex work or exchanged sexual services for financial reasons, which made them a bridge population involved in interactions with diverse populations (Yu et al., 2022). However, a recent study revealed that individuals with a higher education level have a higher enrollment rate in the molecular network of MSM in Chongqing (Bai et al., 2024). Remarkably, among MSM in the high DC+BC group, all three characteristics (junior high school education or below, unemployment, and high baseline viral load) were found to be significant. Therefore, the combination of DC and BC provided insights into the characteristics of influential individuals (high DC) as well as shed light on the characteristics of bridge individuals (high BC) within the network.

Furthermore, risk factors for the high DC group also included CRF07_BC subtype infection and the presence of drug-resistant mutations. Previous studies have indicated that CRF07_BC was the most widely prevalent HIV-1 subtype in China (Wang et al., 2024). In addition, the GD threshold of the CRF07_BC molecular transmission network was significantly lower than that of other subtypes (Weaver et al., 2024), suggesting a higher potential for transmission within populations. This may result in a greater number of connections between individuals in the CRF07_BC network. We had previously reported that the primary drug resistance rate among individuals infected with CRF07_BC was significantly higher compared to other subtypes, and identified drug-resistant molecular clusters involving mutations such as K103N and Q58E within the CRF07_BC molecular network (Zhao et al., 2021b).

Another significant advantage of this study was that it quantified the individual’s HIV transmission risk by calculating their contribution to RHI within the network. In reality, accurately determining the timing of HIV infection to identify the direction of HIV transmission between the linked individuals posed a major challenge. As a result, guidelines often relied on defining high-risk clusters based on the behavioral or demographic characteristics of individuals within the cluster, and recommended intervention measures targeting the entire cluster (Prevention and center, 2019; June 2018). Our study leveraged HIV-1 LAg-Avidity EIA results as well as the timing of HIV diagnosis to infer the direction of HIV transmission among the majority of interconnected PLWH in the network, thereby quantifying their risk of HIV transmission. This analysis significantly improved the resolution for applying molecular networks to guide precise, targeted interventions. Since actual transmission relationship data were unavailable to validate the sensitivity of the inference method—which relied on diagnosis timing and RHI status—there remained a possibility of error in estimating HIV transmission risk using this approach.

This study had several limitations. First, the analysis incorporated only two network parameters—degree and betweenness centrality—along with a limited set of behavioral covariates related to MSM. Future studies should include additional network metrics, such as closeness centrality and eigenvector centrality, as well as a broader range of behavioral factors, to further refine targeted intervention strategies. Second, the findings were based on a molecular transmission network derived from an MSM population in a city in Northeast China. As such, they may not be directly generalizable to other HIV transmission routes, such as injection drug use. Given the potential differences in characteristics and transmission dynamics among PLWH across different risk groups, further research was needed to evaluate the external validity and applicability of these results in other epidemiological settings. Thirdly, it was also important to acknowledge that while we have constructed an HIV transmission network and inferred the directionality of some links within the network, these links did not necessarily represent true transmission relationships.

## Conclusions

The combined utilization of DC and BC can effectively identify individuals at risk of HIV transmission and provide a more comprehensive understanding of their characteristics. This finding provided an effective analysis strategy for leveraging molecular network technology to guide precise targeted intervention.

## Data Availability

The datasets presented in this study can be found in online repositories. The names of the repository/repositories and accession number(s) can be found below: “China HIV gene sequence data platform” (https://nmdc.cn/hiv/) (Sequences numbers: NMDCN0003FE3-NMDCN000678U, NMDCN0003FE7-NMDCN000678T and NMDCN0003FE4-NMDCN0006782) or could be found in online repositories (GenBank accession numbers MW690346, MW690480, MW690493, MW690526, MW690539, PP695701 to PP696626, PP696854 to PP696863 and PX668549 to PX670333).

## Supplementary material

Supplementary Figure 1 Temporal changes of degree centrality **(A)** and betweenness centrality **(B)** and their associated high-risk groups.
