# Supplementary figures and images for: Combining degree centrality and betweenness centrality of molecular networks can effectively pinpoint individuals at high risk of HIV transmission within the network
Source: Front Cell Infect Microbiol. 2026 Jan 19;15:1695049. doi: 10.3389/fcimb.2025.1695049 (PMC12861916; doi:10.3389/fcimb.2025.1695049)

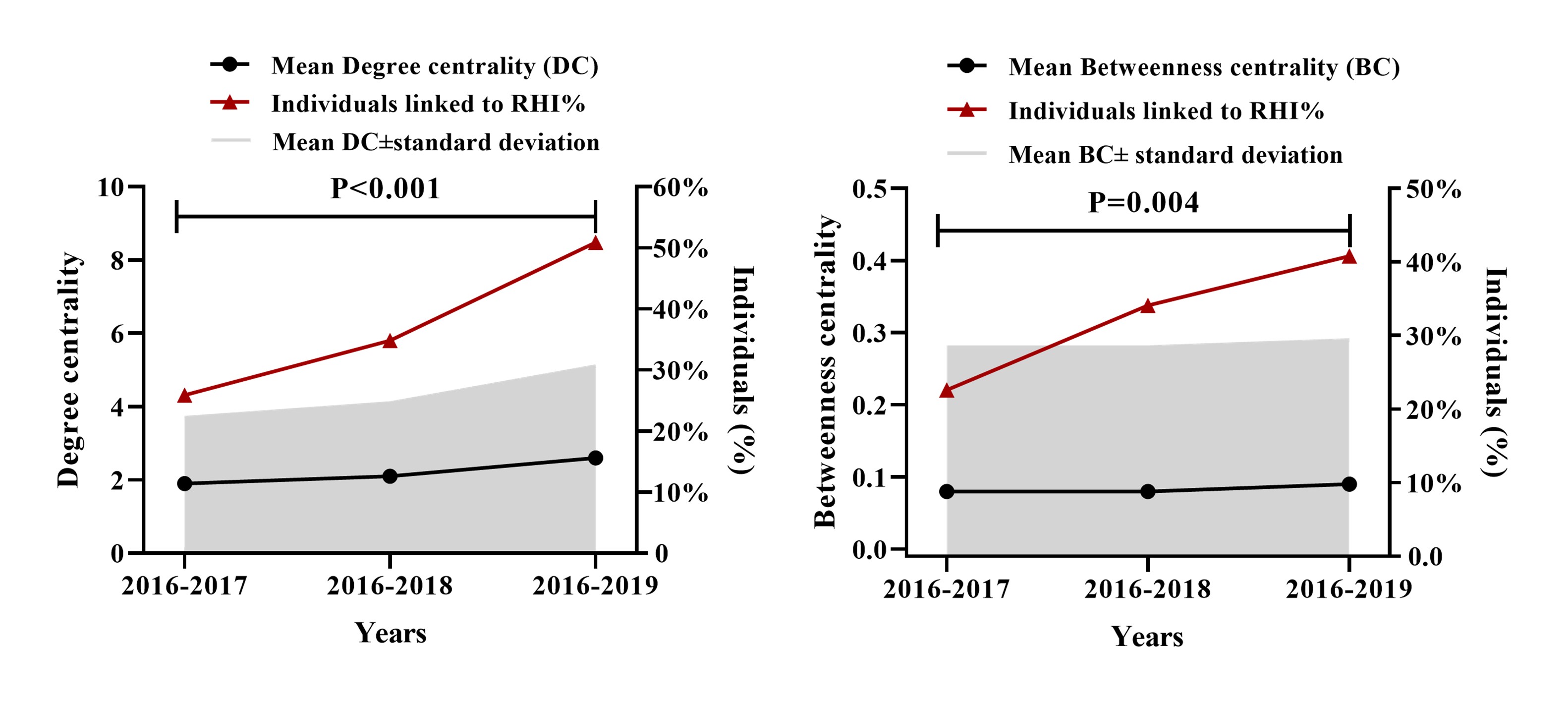

Supplement: Supplementary Figure 1 — Temporal changes of degree centrality (A) and betweenness centrality (B) and their associated high-risk groups. [file Image1.jpeg]
